# Versatility of Chitosan-Based Biomaterials and Their Use as Scaffolds for Tissue Regeneration

**DOI:** 10.1155/2017/8639898

**Published:** 2017-04-16

**Authors:** José Carlos Viana Ribeiro, Rodrigo Silveira Vieira, Iracema Matos Melo, Vilana Maria Adriano Araújo, Vilma Lima

**Affiliations:** ^1^Faculty of Pharmacy, Dentistry and Nursing, Federal University of Ceará, Rua Alexandre Baraúna, No. 949, Rodolfo Teófilo, 60430-160 Fortaleza, CE, Brazil; ^2^Department of Chemical Engineering, Federal University of Ceará, Campus do Pici, Bloco 709, Pici, 60455-760 Fortaleza, CE, Brazil; ^3^Drug Research and Development Center, Department of Physiology and Pharmacology, Federal University of Ceará, Rua Coronel Nunes de Melo, No. 1000, Rodolfo Teófilo, 60430-275 Fortaleza, CE, Brazil

## Abstract

Chitosan is a naturally occurring polysaccharide obtained from chitin, present in abundance in the exoskeletons of crustaceans and insects. It has aroused great interest as a biomaterial for tissue engineering on account of its biocompatibility and biodegradation and its affinity for biomolecules. A significant number of research groups have investigated the application of chitosan as scaffolds for tissue regeneration. However, there is a wide variability in terms of physicochemical characteristics of chitosan used in some studies and its combinations with other biomaterials, making it difficult to compare results and standardize its properties. The current systematic review of literature on the use of chitosan for tissue regeneration consisted of a study of 478 articles in the PubMed database, which resulted, after applying inclusion criteria, in the selection of 61 catalogued, critically analysed works. The results demonstrated the effectiveness of chitosan-based biomaterials in 93.4% of the studies reviewed, whether or not combined with cells and growth factors, in the regeneration of various types of tissues in animals. However, the absence of clinical studies in humans, the inadequate experimental designs, and the lack of information concerning chitosan's characteristics limit the reproducibility and relevance of studies and the clinical applicability of chitosan.

## 1. Introduction

Tissue engineering represents a recent area of multidisciplinary research that applies the knowledge of materials engineering and biology, with the aim of reconstructing or regenerating damaged biological tissue in clinical/pathological situations such as lesions, infections, traumas, and sequelae resulting from tumors or systemic disorders. To this end, it was sought to develop biological substitutes capable of restoring, maintaining, or improving organ and tissue function. Tissue engineering approaches generally involve using a combination of cells, bioactive molecules, and biomaterials. The cells are responsible for the synthesis of the matrix of new tissue. Bioactive molecules, such as cytokines and growth factors, promote cell proliferation, differentiation, and migration. Biomaterials act as a scaffold for the new tissue, providing an environment that favors cell growth and differentiation [1, 2].

**Figure 1:**
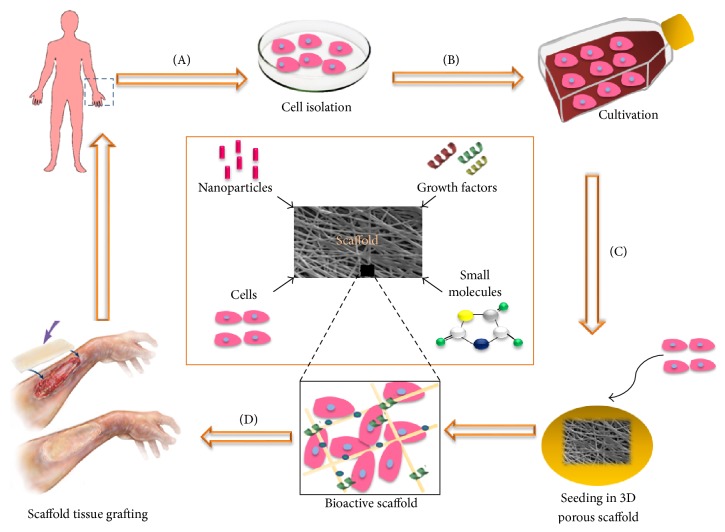
Diagram of the concept of tissue engineering. (A) Cells are isolated from humans or animals, (B) cultivated in vitro, and (C) incorporated into a three-dimensional porous biomaterial (scaffold), together with growth factors, small molecules, and/or micro/nanoparticles. (D) The bioactive scaffold is then grafted onto a tissue lesion, promoting its regeneration. Adapted from Dvir et al. 2011 [12] and Lanza et al. 2014 [2].

All tissue is basically made up of an extracellular matrix (ECM) and cell components. The ECM is the structure upon which the cells establish and organize themselves, providing the environment and architecture specific for cell growth and proliferation, functioning as a reservoir of water, nutrients, and growth factors essential to cells [3, 4]. In this regard, when the regeneration of tissue is desired, the presence is required of a structure that acts as a temporary matrix, mimicking the ECM, allowing cell proliferation and tissue synthesis, until regeneration is complete [5, 6]. This structure, the scaffold, may be defined as a porous, three-dimensional, solid biomaterial that acts as a temporary ECM during tissue regeneration [7]. The biomaterials used as scaffolds in tissue regeneration are of fundamental importance and may determine the success or failure of any tissue engineering approach [8, 9]. Like the ECM, scaffolds must promote vascularization and provide a three-dimensional infrastructure that permits new tissue to form. Also, they must be able to act as a carrier of biologically relevant molecules and, ideally, modulate their biological responses and the regeneration process (Figure 1). Thus, the use of a biomaterial as a scaffold for tissue engineering assumes that it has certain characteristics that make it similar to the extracellular matrix [9–12].

In tissue engineering, the scaffold must have a temporary structure that undergoes gradual biodegradation over a period of time consistent with the speed of new tissue formation, allowing the regenerated tissue to replace it. To this end, it is necessary to possess certain essential characteristics, such as biocompatibility, biodegradability, and mechanical performance and surface properties such as porosity, which favor cell adhesion [7, 10]. The materials most commonly used for tissue regeneration include calcium phosphate ceramics like hydroxyapatite (HA) and beta-tricalcium phosphate (*β*-TCP), synthetic polymers such as polyglycolic acid (PGA) and poly(lactic-co-glycolic) acid (PLGA), and naturally occurring biodegradable polymers such as collagen, hyaluronic acid, silk fibroin, gelatin, and chitosan [5, 13–15].

**Figure 2:**
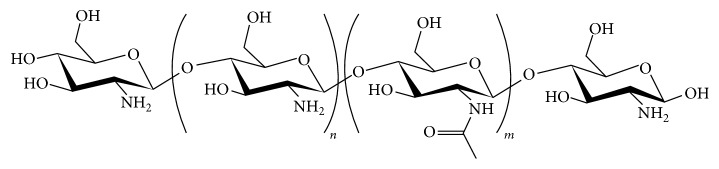
Chemical structure of chitin/chitosan. The index *n* represents the number of repeat units of glucosamine in the chain and *m* the number of repeat units of acetyl-glucosamine in the chain (*n* + *m* indicating the degree of polymerization and *m*/*n* + *m* being the degree of acetylation). When *n* is more than 50%, the polymer is called chitosan. Content of NH_2_ increases its reactivity [16, 25].

Chitosan is a polysaccharide obtained from abundant sources in nature and has been regarded as having great potential for application as a biomaterial for tissue regeneration. It is obtained through the deacetylation of chitin, a polymer found in the exoskeleton of crustaceans, mollusks, and insects and on the cell walls of fungi [16]. Chitosan (CS) has a chemical structure with a linear chain composed of units of* D*-glucosamine and* N*-acetyl-glucosamine joined together via *β* (1–4) glycosidic bonds (Figure 2). In this structure, there are three reactive functional groups: one amine (NH_2_) and two hydroxylic groups (OH). These groupings, particularly the amine, give CS a cationic nature due to the protonation of amino groups (NH_3_+) in acidic medium. This peculiar cationic nature gives CS a great affinity for anionic biomolecules, such as sialic acid, sulfonic acid, and the glycosaminoglycans (GAGs) abundantly present in mucous secretions and extracellular matrix [17, 18]. In fact, CS is considered to be the only natural polysaccharide with this characteristic [19]. By way of ionic interactions, CS has the capacity to bond with mucous tissue, called mucoadhesiveness [10, 20–22]. This property is of great interest to tissue engineering as the possibility of using a scaffold that interacts with the GAGs and tissue's proteoglycans may facilitate the incorporation of cytokines and tissue growth factors, as a large number of these factors have an affinity with GAGs [23, 24].

The singular physicochemical and biological characteristics of CS have prompted numerous studies investigating its application as a scaffold for tissue regeneration [26–31]. Biocompatibility, biodegradability, mucoadhesiveness, absence of toxicity, capacity to form three-dimensional porous structures, ease of handling, and low cost are just some of the benefits mentioned by the authors, which make CS a suitable biomaterial with great potential for application in tissue engineering. Moreover, the reactivity of CS also makes it possible to modify its structure through the substitution of its functional groups, which allows it to be combined with other synthetic or natural polymers, forming the so-called polymer blends, or composites with calcium and phosphate ceramics. These modifications enable the characteristics of CS to be modulated, such as solubility, biodegradability, and mechanical performance, depending upon the type of tissue to be regenerated, which makes it an extremely versatile biomaterial for application in tissue engineering [17, 32–36].

There is, however, a great diversity in the characteristics of CS used in the studies for tissue regeneration, either for the structure of the scaffolds, such as membranes, sponges, films, and nanofibrils, or for the physicochemical parameters of chitosan. The degree of acetylation (DA) and molecular weight (MW), for instance, are the two major chemical properties of CS and influence other physicochemical and biological characteristics, like solubility, crystallinity, mechanical performance, biocompatibility, and biodegradation [19, 37–40]. The lack of standardization in the studies, particularly with regard to the physicochemical characteristics of CS, makes it difficult to establish a consensus as to its applicability as a scaffold for tissue engineering. The present study proposes a systematic review of the literature on the use of chitosan in tissue engineering, with a critical approach to studies on animals and humans, with the aim of establishing a relationship between the methodologies used and results obtained in tissue regeneration, so as to make a contribution to the understanding of the application of this material and to demonstrate its potential use.

## 2. Methodology

For this systematic review, a search was conducted of the* PubMed* electronic database, through the US National Library of Medicine portal (https://www.ncbi.nlm.nih.gov/pubmed/) by two independent researchers, in accordance with the selection criteria. For the search, the following keywords were employed: chitosan, scaffold, tissue engineering, and regeneration. The initial study produced 478 articles. The search was restricted to the last five years and only preclinical studies (with animals) or clinical studies (humans) were used, resulting in 246 articles. After a critical reading of the titles, abstracts, and, where necessary, the articles' methodology, only articles published in English were included. The following exclusion criteria were then applied:

- Literature reviews
- Case studies
- Studies in which CS was not used to evaluate tissue regeneration
- Abstracts or studies where the full content was not accessible

**Figure 3:**
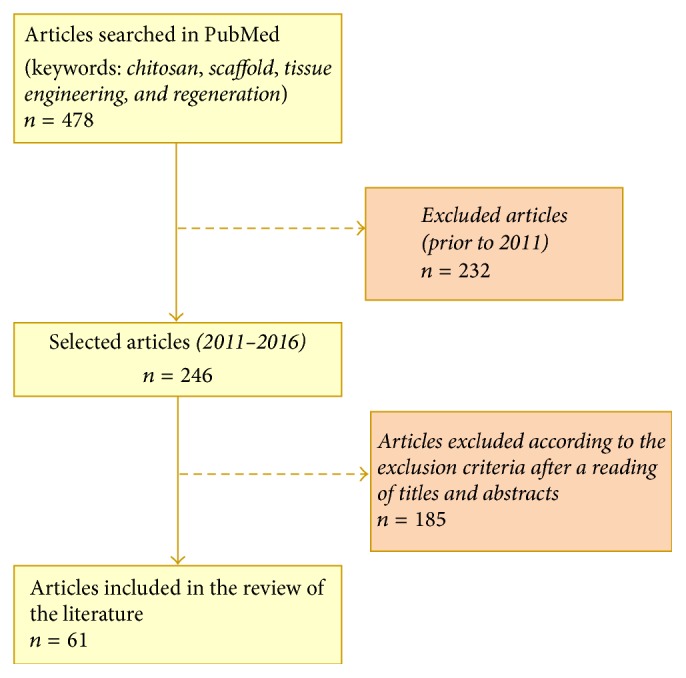
Flowchart of the research study and selection of articles for bibliographical review.

The final selection resulted in 61 articles that were studied, catalogued, and grouped in tables, according to the type of tissues that was involved. A flowchart of the bibliographic research is displayed in Figure 3.

## 3. Results

The selected articles were analysed and the results are shown in Tables 1–5, which list, according to the type of tissues involved, most relevant information such as the scaffold characteristics, main physicochemical properties of CS (when available), combinations with other polymers, incorporation of growth factors and/or cells, the in vitro and in vivo methodology evaluated, the main results in terms of tissue regeneration, and the authors' major conclusions.

Of the 61 studies selected, 60 consisted of preclinical assays using rats (51.6%), rabbits (26.6%), mice (13.3%), dogs (5%), and guinea pigs (3.5%). Only one article demonstrated the effects of CS on human tissue [68].

As far as the analysed tissue types are concerned, 44.3% investigated the action of CS on bone tissue [8, 28, 41–52, 54–62, 80, 85, 86], osteochondral regeneration [80, 85, 86], and bone and vascular [53] or bone and muscular tissues [56]. Moreover, 19.7% of the studies evaluated CS in the regeneration of skin [24, 27, 29, 63–71], 14.7% in nervous tissue [30, 72–79], 11.5% in cartilage [32, 81–84, 87, 88], and 3.3% in periodontal [33, 90] structures. The remaining studies involved regeneration of colorectal [26], mammary [89], tympanic membrane [31], and vascular [34] tissues (1.6% each).

As for the characteristics of the scaffolds, the majority of studies involved CS combined with other biomaterials (85.25%), including HA (16%), collagen (14%), gelatin (10%), silk fibroin (10%), PLGA (8%), multiple combinations between them (20%), or forming other polymer blends (22%). Scaffolds of natural CS were used in 14.75% of the studies [26, 28, 31, 33, 59, 64, 74, 75, 79]. The physicochemical characteristics of CS varied greatly. The molecular weight ranged from 22 [74] to 1,800 [73] kilodaltons (kDa), while the degree of acetylation ranged from 2% [54] to 40% [81]. In almost one-half of the studies reviewed (47.5%), the molecular weight and degree of acetylation of the chitosans used were not specified. When these parameters were mentioned, 46.9% of the chitosans exhibited low molecular weight (up to 150 kDa), 37.5% were of average molecular weight (150 to 700 kDa), and 12.6% had a high molecular weight (in excess of 700 kDa). As for the macrostructure of the scaffolds, these were primarily used in the form of sponges (24.6%), membranes (19.6%), conduits or tubules (13.1%), or even as nanofibrils, hydrogels, microspheres, powder, and paste (11.4%) or were unspecified (31.3%). In 63.9% of the studies, there was an incorporation into the scaffolds of bioactive molecules such as growth factors, genetic factors, peptides, extracellular matrix, or cell components such as stem cells and other cells related to tissue involved in regeneration, or even drugs.

As far as the study outline is concerned, 75.4% used in vitro and in vivo assay methodologies in their investigation of scaffold activities with CS, while in 24.6% of studies only in vivo assays were performed. The in vitro analyses consisted of tests on the cytocompatibility of the scaffolds in cell cultures, evaluating cell adhesion, proliferation, viability, and differentiation. The in vivo assays investigated the effects of scaffolds on tissue regeneration and/or their biocompatibility through macroscopic, radiographic, tomographic, and histological analyses and biomolecular tests such as PCR and immunoassays to detect specific tissue markers involved in regeneration. With regard to the use of comparison groups, 59% of the studies used negative control groups, and 32.8% did not use negative control groups, while in 8.2% of studies the control was not specified in the methodology. In 11.5% of studies, both positive and negative controls were employed, while in 8.2% only positive controls were employed. As for sample size, 21.3% of studies used, in their in vivo assays, samples where *n* was less than 4 sample units; 36.1% had a value of *n* between 4 and 6; in 13.1%  *n* was between 8 and 10 and 13.1% had *n* greater than 10. The sample size was not specified in the methodology in 16.4% of the studies reviewed.

The interrelationship between the scaffolds and their effects on tissue regeneration reveals that, in 93.4% of the studies, scaffolds using CS promoted in vivo or ex vivo tissue regeneration. Only 6.6% of the studies [27, 41, 71, 85] showed no evidence of the scaffolds having a positive influence on tissue regeneration.

## 4. Discussion

Regenerative medicine has been highlighted as one of the most promising fields of scientific research towards which the most recent advances in biological sciences are converging, such as tissue engineering, stem-cell technology, and genetic therapy. The progress achieved in the area of tissue engineering is due, for the most part, to the huge volume of studies using biomaterials that offer potential for regeneration of almost all tissues and organs in the human body. In this context, chitosan has received a great attention from the scientific research literature. Its favorable characteristics as a biomaterial, such as biocompatibility, biodegradability, and, above all, chemical affinity with biological molecules, make it an attractive material for tissue regeneration applications. Also, its chemical reactivity allows its properties to be modulated, making it an extremely versatile material [7, 19].

In the present review, the versatility of CS applications can be observed. It was used in scaffolds for the regeneration of bone, cartilage, skin, nerve, periodontal, vascular, colorectal, mammary, and tympanic membrane tissues. However, though CS is considered to be a biomaterial of great potential for over a decade [16], it is somewhat surprising that none of the studies reviewed in this work has applied CS in clinical trials on human beings. In fact, in the only study where CS was evaluated on human tissue, this was implanted in cutaneous tissue ex vivo or, in other words, removed from individuals who had been subjected to breast or abdomen reduction surgery [68]. This paucity of clinical studies may be explained by researchers' difficulties in obtaining ethical research approval for the use of this material, as there are no regulations for the use of CS as a biomaterial for human tissue regeneration. In the USA, the regulatory agency FDA only approves the use of CS in human beings for the bandaging of skin wounds or hemostatic dressings, while, in other countries such as Japan, Italy, Finland and Brazil, its use is approved as a food supplement and in the cosmetics industry [17, 25, 35].

Most of CS's versatility may be explained by the numerous possibilities of modification in its chemical structure through the substitution of its functional groups. The combination of CS with other natural polymers, such as collagen, gelatin, and silk fibroin, synthetic polymers such as PLGA, and compounds of calcium and phosphate, such as HA, permits an improvement in specific properties like solubility, biodegradation, and mechanical performance of CS as a function of the tissue to be regenerated [10]. In fact, some studies have clearly shown that the association of CS with collagen [42, 44, 68], silk fibroin [73], and HA [45, 57, 58] was capable of promoting greater tissue regeneration than pure CS, as in these studies pure or combined CS scaffolds were implanted, making it possible to establish a comparison between them. However, in the vast majority of studies evaluating CS combined with other biomaterials, the favorable results obtained cannot be attributed to the associations, as pure CS was neither evaluated nor used as a control [8, 30, 32, 41, 46, 48, 49, 53, 54, 62, 63, 67, 71, 76, 77, 80, 81, 83, 86, 88].

Another aspect which merits some attention is the great variability in the chemical characteristics of CS used in the studies, such as molecular weight and degree of deacetylation. As it is a polymer which can be obtained from a variety of sources, CS may actually present a large variation in MW (ranging from 30 to 2000 kDa) and in DA (5 to 46%) resulting from the different processing conditions [7, 21]. However, it should be stressed that these properties drive important physical characteristics of chitosan, such as solubility, crystallinity, absorption of water, and mechanical performance, and also biological characteristics, such as biodegradation, antimicrobial activity, mucoadhesiveness, and biocompatibility [7, 10, 21, 36, 61]. Wu et al. [38] and Zheng et al. [39], for instance, demonstrated that the in vitro bioactivity of CS on macrophages was dependant on its MW. Accordingly, it is probable that these parameters may influence the behavior of CS as a biomaterial for tissue regeneration. Thus, the use of such diverse chitosans, such as MW 22 kDa [74] and 1800 kDa [73], though both studies investigated regeneration in nerve tissue, renders any comparison of results unviable. In addition, the fact that MW and DA have not been specified in 47.5% of the studies raises speculation that the authors have overlooked the significance of these parameters. In fact, the lack of information on the physicochemical characteristics and their effects on biological properties of chitosan-based scaffolds is a matter of concern in the literature, since most of the useful applications of CS are determined by DA, MW, and the nature and fraction of substituents as pendant groups [19]. In the present review, only two studies accounted for the variation of MW in the methodology and interpretation of results. In the study by Cao et al. [49], chitosans of different molecular weights were evaluated in vitro and, based on the results, the 98 kDa CS was selected for the in vivo assay. Denost et al. [26] used layers of CS of different MW to fabricate the scaffolds, due to the different characteristics they presented, corroborating the importance of this parameter on the biological activity of CS.

The use of comparison standards (control groups) is essential when investigating a particular experimental material. It is important to compare its effects with other already familiar or used materials (positive control) or with no treatment at all (negative control). In the present review, only 11.5% of the studies used positive and negative controls to compare with the experimental scaffolds [30, 33, 48, 73, 76, 77, 80]. As many as 20 studies (32.8%) did not use negative controls for comparison. Bearing in mind that in some studies the results showed that tissue regeneration also occurred in negative control groups [31, 41, 48, 65], the absence of a negative control could cast doubt on the relevance of the results obtained with regard to the actual effectiveness of the material being investigated.

Sample size is an important consideration in the design of many studies because of its effect on statistical power. Statistical power is the probability that a statistical test will indicate a significant difference when there truly is one. In this review, some studies used small samples for their in vivo assays, with *n* = 3  [32, 46–48, 61, 65, 68, 81], *n* = 2 [27, 56, 69], and even *n* = 1  [72]. It is important to point out that the value of *n* considered in this study is always the number of animals used for each treatment condition. For instance, the study by Sundaramurthi et al. [65] mentions a sample of 30 animals. However, the animals were subdivided into 5 groups and 2 evaluation periods, totaling 10 groups, resulting in three animals for each condition evaluated (*n* = 3). Simões et al. [72] used 4 rats that were evaluated at 4 different points in time, resulting in just 1 animal per period (*n* = 1). Small samples, such as those mentioned in these studies, may compromise the results obtained, thus diminishing the reliability of the statistical differences found or omitting actual differences that could be observed with an increased sample size [32, 48, 69]. Given the importance of the sample size in the study outline and its influence on the results, it is significant to note that 16.4% of the studies reviewed do not clearly specify this information in their methodologies. The omission of this piece of information makes it difficult to interpret the results and establish a comparison between them.

The overall results concerning tissue regeneration with the use of CS-based scaffolds seem to indicate that they favored the regenerative in vivo process in 93.4% of the works reviewed. Although this information may suggest the effectiveness of CS, it is important to consider that quite a lot of the analysed studies used inadequate experimental designs (such as the absence of proper control groups, small sample size, and lack of comparisons between CS alone or combined with other biomaterials). For this reason, it is not possible to unequivocally confirm that the regeneration was due to the presence of chitosan. However, these results strongly suggest that CS fulfils the function of a scaffolding biomaterial as a reservoir of bioactive factors, such as stem cells and growth factors, showing biocompatibility, biodegradation, adsorption capacity, and gradual release of incorporated factors in most of the studies. Biological activity, with regard to the induction of tissue regeneration, seems to be attributed to the cell components and/or growth factors incorporated to the CS-based scaffolds and not to CS itself. In 83.33% of the studies in which scaffolds were compared, either with or without these components, the results showed a better regenerative performance with the presence of cells and/or growth factors. In other studies, there was no difference in results, neither with nor without bioactive factors [29, 56, 80], or there was no comparison between them [46, 62, 81]. Therefore, it cannot be asserted, from the results presented, that chitosan possesses per se activity that induces tissue regeneration, as some authors suggest [7, 10, 16, 22, 25]. Nevertheless, some studies showed that pure CS, without the addition of bioactive factors, may be effective in promoting regeneration on cutaneous [64], nerve [79], colorectal [26], bone [55], periodontal ligament [33], and tympanic membrane [31] tissues.

## 5. Conclusion

The critical analysis of the studies reviewed confirms the potential for the application of chitosan as a biomaterial in tissue engineering, considering the great versatility of this polymer in the regeneration of various types of tissue in preclinical studies. Scaffolds composed of CS combined with other biomaterials, cells, and growth factors were found to be effective in promoting in vivo tissue regeneration. Nevertheless, the large chemical variability of the CS employed, the omission of precise information about its characteristics, and the methodological limitations of the studies make it difficult to reproduce and to establish a standardization for clinical application. Further studies are required with the aim of defining chitosan's ideal physicochemical characteristics for application with each type of tissue and, thus, propose a protocol for its use in clinical studies in order to confirm its effectiveness in tissue regeneration.

## Figures and Tables

**Table 1:** Studies on CS-based scaffolds for bone tissue regeneration.

Authors	Scaffold type	Study outline	Results	Conclusion
Miranda et al. 2011 [8]	CS (DA: 15%; MW: NA) +GEL (3 : 1 ratio) as membranes (2D) or sponges (3D) crosslinked with glutaraldehyde	In vitro: CC in BMMSCs culture In vivo: 8-week-old Lewis rats had the dental alveoli filled with scaffolds and analysed by HT Sample (in vivo): *n* = 5	Cell proliferation and osteogenic differentiation In vitro In vivo: acute inflammation Bone regeneration Slow biodegradation	CS+GEL sponges demonstrated biocompatibility and potential for application in bone tissue engineering

Danilchenko et al. 2011 [41]	CS (low MW and DA 15–25%) + HA composite sponges (at 15 : 85, 30 : 70, 50 : 50, and 80 : 20 ratios)	In vivo: implantation in tibial defects of 4-month-old rats; HT evaluation, SEM and microhardness of tibias; serum bone-specific alkaline phosphatase (BAP) Control group: tibial defects without scaffolds Sample: *n* = 12	Complete biodegradation after 24 days, promotion of bone regeneration However the amount and speed of newly formed bone tissue were no different statistically from the controls	The scaffolds demonstrated biocompatibility and osteoconductive potential

Niu et al. 2011 [42]	CS microspheres (DA 15–25%; MW: NA viscosity: 200 cps) encapsulated with BMP-2 and incorporated with nHA-COL-PLLA (ratio: NA)	In vitro: CC of MC3T3-E1 mouse osteoblastic cell culture; In vivo: implantation of scaffolds in femoral defects of New Zealand white rabbits; HT and radiographic analyses Control group: nHA-COL-PLLA Sample: not specified	Increased cell activity of osteoblasts on the CS+BMP-2 scaffold Increased radiographic density in the group with CS+BMP-2 and a far better repair than with the controls after 4 weeks	CS microspheres demonstrated great potential for use as BMP-2 matrix carrier for bone regeneration *P* < 0.01 (in vitro)

Costa-Pinto et al. 2012 [43]	CS (DA: NA; MW: NA) combined with PBS (1 : 1 ratio), with or without SC	In vitro: human BMMSCs culture and evaluation of osteogenic differentiation In vivo: implantation of scaffolds in calvarial defects of 7-week-old mice; micro-CT of the bone regeneration Control group: untreated defects Sample: *n* = 6	In vitro: osteogenic proliferation and differentiation on CS scaffolds In vivo: the scaffolds were effective in regenerating calvarium bone tissue (better results in group with SC)	The CS-based scaffolds were shown to be biocompatible and promoted bone regeneration in vivo, particularly in the presence of SC

Hou et al. 2012 [44]	(i) COL sponges (ii) COL sponges with BMP2 (iii) COL sponges with CS microspheres (MW of 90 kDa; DA 5%) and BMP2 (ratio: NA)	In vitro: BMP-2 release tests In vivo: implantation of sponges in radius defects of New Zealand white rabbits; micro-CT and HT evaluations; 3-point bending test of the regenerated bones for mechanical evaluation Control group: normal bone Sample: *n* = 23	In vitro: COL+CS+BMP2 produced a slower, more gradual release up to 35 days In vivo: COL+CS+BMP-2 demonstrated better bone regeneration, complete closure of defects (12 weeks) and greater mechanical performance of newly formed bone tissue	Addition of CS improved release of BMP2, promoted better bone regeneration, and increased mechanical performance of regenerated bone (*P* < 0.05)

Zhang et al. 2012 [45]	CS in gel (DA: NA; MW: NA), either pure or in a composite with nHA (ratio: NA)	In vivo: implantation in defects of the femoral condyle of New Zealand white rabbits; CT, macroscopic, and HT analyses of the defects Control group: untreated defects Sample: *n* = 10; *n* = 3 (control)	The CS+nHA group demonstrated greater bone neoformation than the CS and control groups, and complete repair of the defects after 12 weeks. Pure CS was better than the control	CS+nHA has potential for use in bone regeneration of critical defects, favoring new bone formation when compared to CS alone (*P* < 0.05)

Miranda et al. 2012 [46]	CS (DA 15%; MW; NA) + GEL crosslinked with glutaraldehyde and incorporated with BMMSC (ratio: NA)	In vivo: implantation in fresh tooth sockets of Lewis rat molars; CT, HT, and IHC analyses Control group: contralateral untreated tooth sockets Sample: *n* = 3	CS+GEL+SC group presented greater bone formation after 21 and 35 days, with newly formed bone tissue with a greater level of maturity. There was no control with pure CS	There was greater alveolar bone maturation after extraction with the use of CS+GEL+SC (*P* < 0.05)

Florczyk et al. 2013 [47]	CS (DA: NA; MW: NA) +ALG sponges incorporated with BMMSC or BMP-2 (ratio: NA)	In vitro: CC in BMMSC culture of rats In vivo: implantation of scaffolds in critical calvarial defects of Sprague-Dawley rats; micro-CT, HT, and IHC analyses Control group: untreated defects Sample: *n* = 3	CS+ALG+BMP-2 demonstrated the highest percentage of defect closure, expression of markers, and bone regeneration of all the groups, after 16 weeks All groups showed better results than the control	CS+ALG were biocompatible and permitted osteogenic growth and SC differentiation In vitro and presented osteoconductive properties in vivo (*P* < 0.05)

Jiang et al. 2013 [48]	CS+CMC (1 : 1 ratio) membranes and nHA (0, 20, 40 or 60 wt%). DA: NA; MW: NA	In vitro: CC and osteogenic differentiation in osteoblast cell culture; evaluation of biodegradation In vivo: implantation in long defects in the radius of rabbits; radiographic, micro-CT, and HT analyses Control group: untreated defects Sample *n* = 3	In vitro: CS+CMC showed faster degradation; pure CS degraded more slowly; greater cell proliferation and osteogenic differentiation with CS+CMC+nHA (60% wt) In vivo: there was bone regeneration of defects after 12 weeks even in the control, but with greater bone volume in the CS+CMC+nHA group	Cylindrical/spiral CS+CMC+nHA scaffold demonstrated biomimetic behavior, promoting cell adhesion, proliferation, and differentiation In vitro and bone regeneration in vivo

Jia et al. 2014 [28]	(i) CS sponges (MW 100–300 kDa; DA 6.63%) (ii) CS sponges incorporated with osteogenesis and/or angiogenesis inducing genetic factors (RNA)	In vitro: RNA release tests; osteogenic proliferation and differentiation of rat BMSC In vivo: Implantation of scaffolds in calvarial defects of rats Micro-CT analyses Control groups: pure CS and CS with RNA negative control Sample: not specified	In vitro: CS+ both RNAs exhibited greater cell proliferation and osteogenic differentiation than controls In vivo: CS+ both RNAs promoted increase in area of newly formed bone after 3 months compared to controls	CS sponges impregnated with two RNA factors promoted greater in vitro osteogenesis and angiogenesis and bone regeneration in defects of rat calvarias than pure CS (*P* < 0.05)

Cao et al. 2014 [49]	(i) GEL sponge (Gelfoam®) (ii) Gelfoam with BMP-2 (iii) Gelfoam with sulfonated CS (MW 98 kDa; DA: NA) + BMP-2 (ratio: 1 : 1) (iv) Gelfoam with sulfonated CS + BMP2 in nanoparticles	In vitro: CC, osteogenic, and angiogenic differentiation in culture of human umbilical vein endothelial cells In vivo: implantation in long defects in the radius of 5-mo. New Zealand rabbits; micro-CT, HT, and micro-angiography analyses; 3-point bending test for mechanical testing Control groups: (Gelfoam) and normal bone	In vitro: greater cell proliferation and viability in the groups with CS+BMP (nanoparticles) In vivo: better regeneration and angiogenesis in animals with CS+BMP (nanoparticles); mechanical performance was similar to normal bone	CS+BMP in nanoparticles incorporated in GEL promoted greater neovascularization and bone regeneration In vitro and in vivo than GEL alone or with BMP (*P* < 0.05), showing potential for bone and vascular regeneration

Lee et al. 2014 [50]	CS (MW310 kDa; DA 10%) + HA or nano-HA composites (ratio: NA)	In vitro: CC in cell culture of MC3T3-E1 preosteoblasts In vivo: grafts in segmentary tibial defects of New Zealand rabbits; evaluation via micro-CT and HT Control groups: NA Sample: *n* = 6 to 8	In vitro: CS+nHA demonstrated greater cell proliferation and viability In vivo: better histological and radiographic results with discrete ossification in the nHA+CS group	CS+nHA demonstrated potential for application in bone regeneration

Fernandez et al. 2014 [51]	Composite as a paste of CS (DA: NA; MW: NA) and a bioceramic of *β*TCP+CaO+ZnO (ratio: 60 : 40)	In vivo: implantation of scaffolds in critical calvarial defects of 4-mo. Wistar rats; HT and histomorphometric analyses Control groups: untreated defects Sample: *n* = 4	Scaffolds showed bone regeneration after 40 days, with formation of bone marrow, vessels, and avascular cortical bone and complete closure of the defects by day 60 Control results not specified	CS+*β*TCP+CaO+ZnO promoted osteoinduction and neovascularization of the bone defects, showing potential for bone regeneration

Fan et al. 2014 [52]	Composite sponges of CS (MW 255 kDa; DA 15–25%) + Condroitin sulfphate (ratio: 2 : 1) coated with HA; The sponges were used with or without SC and/or BMP-2	In vitro: CC in adipose-derived SC; BMP-2 release assay In vivo: implantation in critical defects in the jaws of rats; analyses via micro-CT, immunofluorescence, and HT Control groups: NA Sample: *n* = 4	In vivo: greater bone formation in the CS+HA+BMP-2+SC group, with greater expression of collagen and osteocalcin, compared to blank scaffolds	CS+BMP2+CS demonstrated great potential for the regeneration of bone defects, with a synergistic effect of the combination (*P* < 0.05)

Koç et al. 2014 [53]	CS sponges (MW 400 kDa; DA < 15%) + HA (ratio: 9 : 1), whether or not activated with VEGF	In vitro: CC and VEGF secretion in osteoblast culture In vivo: implantation in epigastric fasciovascular flaps of Wistar rats; HT and IHC analyses Control groups: untreated flaps and blank scaffolds Sample: *n* = 6	In vitro: CS+HA+VEGF: greater proliferation of osteoblasts and secretion of VEGF In vivo: CS+HA+VEGF +osteoblasts showed greater neovascularization and ectopic bone formation, at 28 days compared to blank scaffolds Control results not specified	CS+HA+VEGF promoted proliferation of human osteoblasts, induction of ectopic bone formation, and vascular neoformation (*P* < 0.05)

Lai et al. 2015 [54]	Nanofibrous membranes of CS (MW: 100 kDa; DA 2%) and SF (ratio 1 : 1) + nHA (10% or 30%), either with or without stem cells.	In vitro: CC and osteogenic differentiation of BMMSC on CS/SF with or without nHA In vivo: subcutaneous implantation of CS/SF/nHA30%/BMMSC in mice; HT and IHC analyses Control group: acellular scaffold Sample: not specified	In vitro: CS+SF+nHA30% exhibited greater osteogenic differentiation In vivo: only CS+SF+nHA with SC induced formation of ectopic osteoid tissue after 8 weeks	The CS+SF+nHA scaffold favored osteogenic proliferation and differentiation in vitro (*P* < 0.05) and when combined with SC, induced bone formation in vivo

Ghosh et al. 2015 [55]	CS (MW 710 kDa; DA < 10%) crosslinked or otherwise, with citric acid and/or carbo-di-imides	In vitro: CC and osteogenic differentiation in culture of BMSC In vivo: implantation in tibial defects of rabbits; HT analysis of bone regeneration Control groups not specified Sample: not specified	In vitro: crosslinked CS with citric acid demonstrated greater osteogenic adhesion, proliferation and differentiation In vivo: dual crosslinked CS exhibited greater deposition of collagen and bone regeneration after 6 weeks	The dual crosslinked CS scaffold demonstrated greater cytocompatibility in vitro and bone regeneration in vivo (*P* < 0.05)

Caridade et al. 2015 [56]	CS membranes (MW 770 kDa; DA 22%) +ALG, crosslinked with carbo-di-imides and incorporated or otherwise with BMP-2 (ratio: NA)	In vitro: CC and myogenic and osteogenic differentiation In vivo: implantation in subcutaneous tissue of mice and evaluation via micro-CT Control groups: not specified Sample: *n* = 2	In vitro: CS+BMP-2 induced osteogenic differentiation and release of BMP-2 In vivo: only the most crosslinked membranes were capable of inducing osteogenesis at 52 days	Crosslinked CS+BMP-2 have potential for use as a periosteum substitute for bone regeneration

Frohbergh et al. 2015 [57]	Microfibers of genipin crosslinked CS (DA 15–25%; medium MW), with or without nHA and SC (ratio: NA)	In vitro: CC and osteogenic differentiation in murine MSC culture In vivo: implantation in calvarial defects of 4–6-w mice; HT and micro-CT analyses Control group: untreated defects Sample: *n* = 4	In vitro: CS+nHA produced twice the osteogenic differentiation of CS In vivo: CS+nHA+SC exhibited greater bone neoformation than any of the others, after 3 months	CS crosslinked with genipin has potential for use in bone regeneration; addition of nHA and stem cells increased bone regeneration in vivo (*P* < 0.01)

Dhivya et al. 2015 [58]	Hydrogels of CS-Zn (DA: NA; MW: NA)+*β*-glycerophosphate + nHA (ratio: 8 : 1; 1) or without nHA	In vitro: cell proliferation and differentiation in mouse MSC culture In vivo: insertion into tibial defects of Wistar rats; radiographic and HT analyses Control group: untreated defects Sample: not specified	In vitro: scaffolds favored osteoblast proliferation and differentiation In vivo: greater mineralization and formation of collagen after 14 days in scaffolds with nHA	CS-Zn + *β*-glycerophosphate demonstrated bone regeneration potential; addition of hydroxyapatite promoted and accelerated bone formation (*P* < 0.05)

D'Mello et al. 2015 [59]	Sponges of CS (MW: 110 to 150 kDa; DA: NA), whether or not incorporated with copper sulfate (ratio: NA)	In vivo: implantation in calvarial defects of 14-week-old Fisher rats; analyses via micro-CT and HT Control groups: untreated defects Sample: *n* = 2 (control *n* = 3)	CS + copper exhibited greater bone neoformation than pure CS or control, both via micro-CT and via histological analyses	CS + copper has great potential for application in bone regeneration and promoted bone regeneration in vivo (*P* < 0.05)

Ji et al. 2015 [60]	3D disks of CS (low MW; DA: NA) +GEL with spherical or cylindrical nHA (ratio: 1 : 1 : 3) with or without SC.	In vitro: morphology, osteogenic proliferation, and differentiation of human gingival fibroblast-derived induced pluripotent SC In vivo: implantation of scaffolds with or without SC in subcutaneous tissue of mice; HT and IHC analyses of ectopic bone-like tissue formation Control group: not specified Sample: *n* = 12	In vitro: scaffolds with spherical nHA demonstrated greater osteogenic proliferation and differentiation (*P* < 0.01) In vivo: CS+GEL+ nHA+SC showed greater bone-like tissue formation than acellular scaffolds (*P* < 0.01); spherical nHA induced thicker bone-like formation after 12 weeks (*P* < 0.05)	CS+GEL+spherical nHA combined with pluripotent human cells induced ectopic bone-like tissue formation and represent an innovative approach with the potential for application in bone tissue engineering

Shalumon et al. 2015 [61]	Nanofibrous membranes of CS (MW 100 kDa; DA 2%) +SF+nHA+BMP2, whether or not impregnated with SC (ratio: NA)	In vitro: osteogenic proliferation and differentiation of MSC; BMP-2 release test In vivo: implantation of scaffolds with or without MSC in subcutaneous tissue of 6–8-week-old mice; HT and IHC analyses after 4 and 8 weeks Control groups: not specified Sample: *n* = 3	In vitro: BMP-2 increased osteogenic differentiation of MSC on CS+SF and CS+SF+nHA scaffolds In vivo: cellular or acellular scaffolds were capable of inducing formation of ectopic bone-like tissue, with greater intensity when SC was present	CS+SF+nHA scaffolds with BMP-2 induced greater osteogenic differentiation In vitro (*P* < 0.05) and showed great potential for application in bone regeneration in vivo

Xie et al. 2016 [62]	Nanofibers of CS (DA < 15%; MW: NA)+HA (ratio: 7 : 3) and/or COL+SC	In vitro: CC and osteogenic differentiation of induced pluripotent SC+ MSC In vivo: implantation scaffolds with or without SC in critical calvarial defects of 6-week-old mice; HT analysis (4, 6, and 8 weeks) and tomographic analysis (6 weeks) Control group: untreated defects, pure CS, and TCP Sample: *n* = 2 (histology); *n* = 6 (CT)	In vitro: CS+HA+COL promoted greater osteogenic differentiation than CS, CS+HA, and TCP In vivo: CS+HA+COL with SC promoted greater bone neoformation, via CT and histology, with complete regeneration of defects	CS+COL+HA with stem cells promoted effective bone neoformation in vitro and in vivo, with better results than controls (*P* < 0.01), showing potential for bone regeneration in clinical applications

ALG: alginate; BAP: bone alkaline phosphatase; BMMSCs: bone marrow mesenchymal stem cells; BMP2: type 2 morphogenetic bone protein; CC: cytocompatibility; CMC: carboxymethyl cellulose; COL: collagen; CS: chitosan; CT/micro-CT: computed tomography/micro-computed tomography; DA: degree of acetylation; GEL: gelatin; HA/nHA: hydroxyapatite/nanohydroxyapatite; HT: histological; kDa: kilodaltons; MSC: mesenchymal stem cells; MW: molecular weight; NA: not available; PLLA: poly-L-lactate; SC/BMSC: stem cells/bone marrow stem cells; SF: silk fibroin; TCP/*β*TCP: tricalcium phosphate.

**Table 2:** Studies on CS-based scaffolds for cutaneous tissue regeneration.

Authors	Scaffold type	Study outline	Results	Conclusion
Guo et al. 2011 [63]	Bilayer CS (DA 15–25%; MW: 100–170 kDa) +COL membranes (ratio NA) impregnated or not with TMC (DD 90%) and VEGF (plasmid-DNA encoded)	In vitro: CC in HUVEC culture In vivo: implantation of membranes on burn skin lesions on the backs of guinea pigs. HT, PCR, and *Western-blot* analyses Control groups: blank scaffolds and CS/COL/TMC/pDNA without VEGF Sample: not specified	Greater cell viability and VEGF expression in scaffolds with TMC/pDNA-VEGF than the controls in vitro Greater angiogenesis, VEGF expression and better repair of wounds with TMC/pDNA-VEGF scaffolds in vivo	CS+COL impregnated with TMC and VEGF promoted angiogenesis and dermal regeneration (*P* < 0.05), showing potential for use in the regeneration of epithelial lesions

Tchemtchoua et al. 2011 [64]	Films, sponges, and CS nanofibrils (DA 16% and MW 67 kDa)	In vitro: CC in a culture of fibroblasts, keratinocytes, and endothelial cells In vivo: implantation in the subcutaneous tissue (back skin) of 10-week-old mice (biocompatibility) and in skin defects; HT evaluation Control: untreated defects Sample: *n* = 10	In vitro: greater adhesion, cell proliferation, and differentiation with nanofibrillar CS In vivo: greater biocompatibility and faster regeneration of wounds with nanofibrillar CS; CS sponges caused foreign body reaction	The authors conclude that the nanofibrillar form has advantages over the others, being more biocompatible and effective in regeneration of the skin

Sundaramurthi et al. 2012 [65]	CS (DA 15%; MW: NA) in nanofibrils or films (crosslinked with glutaraldehyde), in combination with PVA and RSPO1 (50 ng)	In vitro: CC in fibroblast cell culture; evaluation by RT-PCR In vivo: implantation on skin wounds on rats' backs; macroscopic and HT evaluation Control groups: untreated wounds (negative), Bactigras® (positive) Sample: *n* = 3	In vitro: greater cell adhesion and proliferation in nanofibrillar CS+PVA group (*P* < 0.05) In vivo: complete macroscopic regeneration after 2 weeks and better histopathological results in the CS+PVA+RSPO1 group (*P* < 0.05)	CS+PVA demonstrated good results as carrier of the growth factor, constituting a biocompatible biomaterial with potential for application as a skin substitute

Veleirinho et al. 2012 [66]	CS (medium MW; DA: NA) combined with the polymer PHBV (ratios: 2 : 3 and 1 : 4)	In vitro: CC in a culture of fibroblast cells of mouse; In vivo: implantation of scaffolds and a commercial biomaterial as a control in skin wounds on the backs of 2-month-old rats; macroscopic and HT evaluation of regeneration. Sample: not specified	In vitro: cell viability and proliferation with CS+PHBV (1 : 4) similar to the control (*P* > 0.05) In vivo: greater organization and maturation of the epithelial tissue with CS+PHBV (1 : 4); lower occurrence of inflammatory infiltrate with CS+PHBV (2 : 3)	CS+PHBV has potential for promoting skin regeneration, with biocompatibility in vitro

Wang et al. 2013 [67]	CS membranes (MW 100 to 171 kDa; DA 15%) + COL and PLGA (ratio: NA)	In vivo: implantation of the scaffolds, with or without PLGA, on skin defects in backs of 2-month-old rats; macroscopic, HT, IHC, PCE analyses and tensile strength tests. Sample*: n* = 12	CS+COL+PLGA scaffolds demonstrated better healing and greater expression of IHC and PCR markers and higher mechanical performance (*P* < 0.05)	CS+COL scaffolds reinforced with PLGA demonstrated acceleration of angiogenesis and better skin regeneration than CS+COL (*P* < 0.05)

Sarkar et al. 2013 [68]	Crosslinked CS membranes (MW 71 kDa; DA < 10%) whether or not combined with COL	In vitro: CC in culture of fibroblasts and keratinocytes. In vivo: implantation in human skin defects ex vivo; HT analyses. Control groups: not specified Sample: *n* = 3	In vitro: CS+COL demonstrated better cell adhesion, proliferation, and viability In vivo: CS+COL promoted partial reepithelialization with migration after 14 days; pure CS did not promote regeneration	CS+COL scaffold promoted better regeneration of skin wounds than pure CS scaffolds (*P* < 0.05)

Zeinali et al. 2014 [24]	CS membranes (medium MW; DA 15–25%) crosslinked with PHBV, with or without SC (2 × 10^6^)	In vitro: CC in umbilical cord SC culture; In vivo: implantation in skin defects on 4–8-week-old rats' backs; HT and IHC analyses. Control not specified. Sample: *n* = 10	In vitro: CS+PHBV showed greater cell proliferation and viability In vivo: greater regeneration of cutaneous tissue with CS+PHBV+SC Statistical analysis not performed	CS+PHBV added to stem cells was capable of regenerating full thickness skin defects in rats

Guo et al. 2014 [69]	Bilayer CS (MW 100–170 kDa; DA 15–25%) +COL and silicone membranes (ratio: NA)	In vivo: implantation of scaffolds in excisional or burnt skin lesions in guinea pigs; HT, IHC and IF evaluations; c Control group: commercial bandage; sample: *n* = 2	CS+COL produced results inferior to the control in the regeneration of burn lesions (*P* < 0.05) There was no significant difference with excisional lesions (*P* > 0.05)	CS and collagen demonstrated effectiveness similar to the commercial product in the regeneration of skin damaged by excisional wounds

Revi et al. 2014 [29]	CS sponges (MW 354 kDa; DA 14%) impregnated or not with keratinocytes and fibroblasts	In vivo: implantation of scaffolds or unspecified commercial product (positive control) in dorsal skin lesions of rabbits; HT and IHC analyses; no negative control. Sample: *n* = 6	CS scaffolds with or without cells exhibited slower complete reepithelization of lesions (28 days) than commercial product (14 days) (*P* = 0.02 compared to CS without cells and *P* = 0.03 compared to CS with cells)	CS sponges combined with dermal cells showed potential for application in the regeneration of complete skin defects

Han et al. 2014 [27]	CS sponges + GEL (ratio NA) incorporated with antibacterial drugs. MW and DA not reported. Drugs not reported	In vitro: CC in culture of skin fibroblasts); porosity, water absorption and biodegradation tests; In vivo: implantation of scaffolds in skin lesions on rabbits' backs; HT analysis of biocompatibility; no negative control. Sample: *n* = 4	In vitro: CS+GEL demonstrated adequate CC In vivo: inflammatory infiltrate present within 15 days, being lower in sponges with antimicrobials; there was no lesion regeneration Statistical analysis was not performed	CS+GEL exhibited adequate physicochemical properties and cytocompatibility in vitro, but induced inflammation in vivo

Ahamed et al. 2015 [70]	CS+ cellulose (ratio NA), incorporated with nanoparticles of silver, with or without gentamicin. MW and DA not reported	In vivo: implantation in skin lesions in the backs of Wistar rats; macroscopic, biochemical and planimetric analyses. Controls: sterile cotton gauze dipped with gentamicin or standard soframycin ointment. Sample: *n* = 3	Scaffolds with or without gentamicin did not exhibit any difference between one another but were better than controls The healing was complete after 25 days *P* not reported	CS + cellulose was effective in the regeneration of skin wounds

Wang et al. 2016 [71]	COL+CS (DA < 15%; MW 106–171 kDa) + PLGA + PUR (ratio NA)	In vivo: implantation in skin lesions on the backs of 2-month-old Sprague-Dawley rats; SEM, HT and IHC analyses; tensile strength tests; Control groups; commercial membrane (COL + silicon); Sample: *n* = 12	COL+CS+PLGA+PUR showed greater expression of angiogenesis markers, better regeneration of cutaneous tissue wounds and better mechanical performance than commercial membrane used as control	COL+CS+PLGA+PUR membranes promoted better regeneration of skin defects in comparison with commercial membrane (*P* < 0.05)

CC: cytocompatibility; COL: collagen; CS: chitosan; DA: degree of acetylation; HT: histological; IHC: immunohistochemical; kDa: kilodaltons; MW: molecular weight; PCE: polycaprolactone-polyethylene glycol polymer; PHBV: poly(3-hydroxybutyrate-co-3-hydroxyvalerate; PLGA: polylactic-co-glycolic acid; PUR: polyurethane; PVA: polyvinyl-alcohol; RT-PCR: real time-polymerase chain reaction; RSPO1: R-spondin-1 angiogenesis growth factor; TMC: trimethyl chitosan chloride; VEGF: vascular endothelial growth factor.

**Table 3:** Studies on CS-based scaffolds for nerve tissue regeneration.

Authors	Scaffold type	Study outline	Results	Conclusion
Simões et al. 2011 [72]	High MW CS membranes crosslinked with GPTMS. (DA: NA; ratio: NA)	In vitro: CC in neuroblastoma clone cell culture (N1E-115); fluorescence microscopy for intracellular Ca++ In vivo: HT analysis of the subcutaneous tissue in adult Wistar rats. No control group *Sample* (in vivo): *n* = 4	CS membranes promoted cell adhesion and differentiation in vitro In vivo: slight to intense chronic inflammation was observed in HT analysis Presence of fibrous capsule	Authors concluded that CS membranes demonstrated biocompatibility and potential for use in the regeneration of nerve tissue However, the presence of chronic inflammation and fibrous capsules contradict the conclusion (*P* < 0.05)

Wei et al. 2011 [73]	CS (MW 1800 kDa; DA 6.5%) + SF films (ratios: 50 : 50 or 70 : 30) impregnated with SC	In vitro: CC in SC culture and Schwann cells In vivo: implantation of scaffolds in lesions of the sciatic nerve in adult Sprague-Dawley rats; functional and HT evaluation Control group: nongrafted rats Sample: *n* = 8	In vitro: greater adhesion and proliferation with CS and SF scaffolds when compared to pure CS In vivo: Greater functional recovery and tissue regeneration in the groups with CS+SF impregnated with SC	CS+SF impregnated with SC promoted better regeneration in sciatic nerve lesions and lower proliferation of fibrous scar tissue (*P* < 0.05)

Chen et al. 2011 [74]	CS conduits (DA 7.7%; MW 22 kDa) whether or not impregnated with BMMSC	In vivo: implantation of conduits in spinal cord defects in adult Sprague-Dawley rats; functional evaluation and electromyography; HT and IHC analyses Control group: untreated defects Sample: *n* = 15 Control: *n* = 10	Better motor and electromyographic response with CS+BMSC; better macroscopic and HT regeneration of defects filled with scaffolds with SC	CS +SC scaffolds were capable of promoting axonal regeneration, remyelination, and functional recovery after sectioning of spinal cord (*P* < 0.05)

Liao et al. 2012 [75]	CS in the form of conduits (DA 15–25%; MW: NA), either with or without SC impregnation	In vivo: implantation of scaffolds in sciatic nerve defects in adult Sprague-Dawley rats; evaluation of repair through magnetic resonance, functional evaluation, and HT analyses. Control group not specified Sample: *n* = 18	Nerves implanted with scaffolds impregnated with MSC demonstrated better functional recovery and better magnetic resonance results than acellular scaffolds	CS impregnated with SC promoted regeneration of nerve tissue; magnetic resonance was effective for evaluating regeneration of the sciatic nerve (*P* < 0.05)

Xue et al. 2012 [76]	CS+PLGA (ratio NA) in the form of tubes, whether or not impregnated with SC (DA: NA; MW: NA)	In vivo: grafting of conduits on to sciatic nerve defects in adult Beagle dogs; functional and electroneuromyographic evaluations and neuron count; morphometric analysis and HT analysis of associated muscles Control groups: nongrafted and autogenous grafted defects Sample: *n* = 5	Better functional recovery in CS+PLGA+SC group; remyelination and recovery of nerve diameter; histologically, greater regeneration in the autogenic and CS+PLA+SC groups	CS+PLGA scaffold, either with or without stem cells, favored regeneration of extensive sciatic nerve lesion and showed viability of carrying out a clinical study with this material (*P* < 0.05)

Xiao et al. 2013 [77]	CS+COL (ratio: 1 : 4) in the form of conduits, whether or not combined with RGD peptide (DA: NA; MW: NA)	In vivo: implantation of scaffolds in segmental defects of adult Sprague-Dawley rats sciatic nerves; functional evaluation via electroneuromyography, neuron markers, and histology Control groups: untreated defects and autogenous grafted defects Sample: *n* = 8	CS+COL scaffolds showed better functional recovery than negative control; CS+COL+RGD showed greater management of nerve stimuli than negative control, but lower than the autogenous control. Scaffolds demonstrated greater tissue regeneration than negative control but less than the positive control	CS+COL+RGD was capable of accelerating the regeneration of the sciatic nerve, obtaining satisfactory results in 2 months (*P* < 0.05)

Biazar and Keshel 2013 [78]	CS (medium MW; DA 15–25%) in the form of conduits, whether or not crosslinked with PHBV	In vitro: CC in Schwann cell culture In vivo: implantation of scaffolds in sciatic nerve defects of 4–8-week-old Wistar rats; macroscopic and microscopic analyses via HT and IHC Control groups: untreated defects and autogenous grafted defects Sample: *n* = 5	In vitro: CS+PHBV was found to exhibit greater cell viability and proliferation In vivo: CS (crosslinked or not with PHBV), produced regeneration results far superior to the negative control, though inferior to the autogenous control	CS+PHBV demonstrated capacity to regenerate lesions of the sciatic nerve in rats (*P* < 0.05), having potential for application in tissue engineering and clinical studies

Gu et al. 2014 [30]	CS+SF (ratio NA) in the form of conduits impregnated with EMC (DA: NA; MW: NA)	In vitro: isolation of Schwann cell EMC derived from rats In vivo: implantation in sciatic nerve defects in adult Sprague-Dawley rats; HT and IHC analyses; electrophysiological tests Control group: acellular xenogeneic nerve graft Sample: not specified	In vivo: better nerve tissue regeneration and density in the CS+SF+EMC group after 12 weeks. The electrophysiological tests got a response in all groups, though to a lesser extent in the CS+SF group	The CS+SF+EMC scaffold was effective in regenerating nerve tissue (*P* < 0.05)

Wang et al. 2016 [79]	CS conduits (DD 92.3%; MW 250 kDa) or chitooligosaccharides (COS) in silicon conduits	In vitro: CS biodegradation and CC in Schwann cell culture In vivo: implantation of scaffolds in lesions of the sciatic nerves of adult Sprague-Dawley rats; HT and IHC analyses Control: saline group Sample: not specified	In vitro: COS promoted greater cell proliferation and differentiation In vivo: greater expression of nerve cell markers in the chitooligosaccharide groups	Chitooligosaccharides promote nerve cell proliferation and differentiation, stimulating regeneration of nerve tissue (*P* < 0.05/*P* < 0.01)

BMMSCs: bone marrow mesenchymal stem cells; CC: cytocompatibility; COL: collagen; COS: chitooligosaccharides; CS: chitosan; DA: degree of acetylation; ECM: extracellular matrix; GPTMS: glycidoxypropyltrimethoxysilane; HT: histological; IHC: immunohistochemical; kDa: kilodaltons; MW: molecular weight; PLGA: polylactic-co-glycolic acid; RGD: cell-adhesive peptide; SC/BMSC: stem cells/bone marrow stem cells; SF: silk fibroin.

**Table 4:** Studies on CS-based scaffolds for cartilage tissue regeneration.

Authors	Scaffold type	Study outline	Results	Conclusion
Chen et al. 2011 [80]	CS sponges (DA 15%; MW 400 kDa) +HA+GEL (ratio NA) whether or not activated by growth factors (BMP-2 and TGF)	In vitro: CC and cell differentiation in MSC culture In vivo: implantation of sponges in osteochondral patellar defects of 4-month-old New Zealand white rabbits; HT and IHC evaluations Control group: DNA-free composite osteochondral graft Sample: *n* = 5 Control: *n* = 4	Growth and osteochondral differentiation in vitro were observed In vivo: greater osteochondral tissue neoformation with the scaffolds groups, with or without growth factors when compared to the control	CS sponges + HA+GEL with TGF and BMP-2 promoted greater cell growth and bone and cartilage tissue regeneration (*P* < 0.05)

Whu et al. 2013 [81]	CS (MW 65 kDa; DA 40%) +GEL (ratios 5 : 0, 4 : 1, 3 : 2, 1 : 1, 2 : 3, 1 : 4, or 0 : 5) in films and sponges, either crosslinked or not with carbodiimide	In vitro: CC in culture of chondrocytes with scaffolds In vivo: implantation of crosslinked scaffolds in cartilage defects in rabbits feet; HT and IHC analyses Control group: untreated defects Sample: *n* = 3	In vitro: greater cell proliferation and viability with crosslinked CS+GEL scaffolds In vivo: greater regeneration of cartilage with CS+GEL impregnated with chondrocytes after 1 month There was no comparison with pure CS or with absence of chondrocytes	The authors conclude that carbodiimide crosslinked CS+GEL scaffold demonstrated potential for cartilage regeneration (*P* < 0.05)

Zhang et al. 2013 [82]	Sponges of CS (MW 40 kDa; DA: NA) +PLGA (ratio 1 : 1), either with or without incorporation of SC	In vitro: CC in adipose-derived stem-cell culture, in chondrogenic medium In vivo: implantation of scaffolds, with or without SC, in articular defects in 4-month-old New Zealand rabbits knees; HT, IHC, and biomechanical assays (compressive modulus and cytonano-indentation) Control group: scaffold alone Sample: *n* = 5	In vitro: CS+PLGA favored chondrogenic adhesion, proliferation, and differentiation In vivo: CS+PLGA+SC promoted greater regeneration of the defects and maintenance of subchondral bone, after 12 weeks, and greater mechanical performance than scaffolds without SC	CS+PLGA+SC scaffolds were capable of regenerating the full thickness of the cartilage defects in 12 weeks (*P* < 0.05)

Deng et al. 2013 [83]	Sponges of CS (DD: NA; MW: NA) +SF (ratio 1 : 1); DA: NA; MW: NA incorporated or not with SC	In vitro: CC in BMMSC culture In vivo: filling of defects in the cartilage of 2-3- month-old New Zealand rabbit knees with scaffolds, with or without SC; HT and IHC analyses Control group: untreated group Sample: *n* = 6	In vitro: CS scaffolds promoted chondrogenic differentiation In vivo: the CS+SF+SC scaffold promoted almost complete repair of the defects and positive HT and IHC results; CS+SF scaffold demonstrated better results than the control, but not as good as in the group with SC	CS+SF scaffold showed itself to be effective as SC carrier and capable of being used in the regeneration of cartilage tissue (*P* < 0.05)

Wu et al. 2014 [84]	Sponges of pure CS (DA: NA; MW: NA) or combined with fibrin (ratio NA), whether or not incorporated with SC	In vitro: CC and chondrogenic differentiation in SC culture from synovial fluid In vivo: implantation in defects in 2-week-old nude mice TMJ disc; HT, IHC, and PCR analyses Control group: cell-free chitosan/fibrin scaffold Sample: *n* = 6	In vitro: CS + fibrin exhibited greater chondrogenic adhesion, proliferation, and differentiation In vivo: CS+SC and CS+fibrin+SC induced greater regeneration than acellular scaffolds at 4 weeks	CS + fibrin scaffold with TMJ-derived stem cells demonstrated regenerative capacity for the treatment of TMJ disc perforations (*P* < 0.05)

Cheng et al. 2014 [32]	Membranes of CS (DA ≤ 10%; MW: 200–500 kDa) +PLGA (ratio 75 : 25), whether or not impregnated with chondrocytes	In vitro: CC and chondrogenic differentiation inBMMSC culture In vivo: implantation in cartilage defects of 2-month-old New Zealand rabbit ears; macroscopic and HT analyses Control group: untreated defects Sample: *n* = 3	CS+PLGA + chondrocytes demonstrated complete and homogeneous regeneration after 18 weeks, with the formation of mature cartilage tissue; acellular scaffold and control group exhibited fibrosis	CS+PLGA impregnated with chondrocytes were capable of regenerating the cartilage tissue (*P* not reported)

Ravanetti et al. 2015 [85]	CS+ raffinose (DA: NA; MW: NA; ratio: NA)	In vivo: implantation of scaffolds in osteochondral defects in the scapula of New Zealand white rabbits; macroscopic and HT analyses; negative control Control group: untreated defects Sample: *n* = 9	CS + raffinose did not promote regeneration of defects histologically or macroscopically and induced inflammation and formation of fibrous capsule, after 4 weeks	The authors conclude that CS + raffinose has limitations and that further studies are needed before application. *P* > 0.05

Ravindran et al. 2015 [86]	CS+COL (1 : 1), with or without SC and ECM (DA: NA; MW: NA)	In vitro: culture of MSC in osteogenic and chondrogenic medium In vivo: implantation in the subcutaneous tissue of mice; HT, IHC, and magnetic resonance analyses Control group: scaffolds without ECM Sample: not specified	In vitro:ECM scaffolds induced chondrogenic differentiation In vivo: CS+COL+SC+ECM presented expression of chondrogenic differentiation markers after 2 weeks; with magnetic resonance, newly formed tissue similar to native cartilage was observed after 8 weeks	The CS+COL+SC+ECM scaffold demonstrated efficiency in the regeneration of cartilage and bone tissue *P* < 0.01

Meng et al. 2015 [87]	CS hydrogel, either with or without DBM particles, E7 peptide (P7), and SC (DA = NA; MW = NA; ratio = NA)	In vitro: CC and chondrogenic differentiation in culture of BMSC; compression strength and elastic modulus tests In vivo: HT and IHC analyses of subcutaneous tissue of nude mice after 4 weeks Control group: pure CS scaffolds and composite scaffolds of DBM and CS; Sample: *n* = 5	In vitro: greater cell proliferation and differentiation with CS+DBM+P7 Preparation of DBM particles might influence the mechanical properties of scaffolds and cell proliferation In vivo: CS+DBM+P7+SC produced greater cartilage tissue formation than pure CS or with DBM; no negative control	CS+DBM+P7 hydrogel combined with mesenchymal stem cells has potential for regeneration of cartilage tissue. *P* < 0.05

Zhang et al. 2015 [88]	CS sponges (MW 40 kDa; DA < 5%) +PLGA, with or without SC; ratio: NA; average viscosity	In vitro: CC and chondrogenic differentiation in culture of SC In vivo: implantation in cartilage defects in 4-month-old New Zealand rabbit knees; macroscopic, HT and IHC analyses; negative control not specified Control group: adherent ASC/scaffold complexes Sample: *n* = 5	In vitro: CS+PLGA+SC demonstrated chondrogenic differentiation In vivo: the scaffold promoted new formation of cartilage similar to hyaline, both histologically and via biomechanical evaluation, after 6 and 12 weeks	CS+PLGA sponges incorporated with aggregated stem cells represents a promising technique in tissue regeneration *P* < 0.05

CC: cytocompatibility; COL: collagen; CS: chitosan; DA: degree of acetylation; DBM: Demineralized bone matrix; ECM: extracellular matrix; HT: histological; IHC: immunohistochemical; kDa: kilodaltons; MW: molecular weight; PCR: polymerase chain reaction; PLGA: polylactic-co-glycolic acid; SC/BMSC: stem cells/bone marrow stem cells; TMJ: temporomandibular joint.

**Table 5:** Studies on CS-based scaffolds for regeneration of diverse tissues.

Authors	Tissue type	Scaffold type	Study outline	Results	Conclusion
Gupta et al. 2011 [89]	Mammary tissue	CS+SF (ratio NA) scaffolds impregnated with emodin (antitumor drug) MW and DA not reported	In vitro: breast cancer cell culture; evaluation of cell growth and viability In vivo: implantation in breast tumor tissue in nude rats; HT analyses Control: flap tissue without scaffolds Sample: *n* = 8 Control *n* = 7	In vitro: cell proliferation with no statistical difference in the control and CS+SF without drugs (*P* > 0.05) In vivo: there was a reduction in cancerous cells only in the CS+SF group with emodin (*P* < 0.05) Tissue regeneration was similar between scaffolds with or without emodin (*P* > 0.05)	CS+SF scaffolds were effective in absorption, release, and pharmacological activity of the therapeutic agent and in the regeneration of the tissue defect

Seonwoo et al. 2013 [31]	Tympanic membrane	CS membranes (MW 200 kDa; DA 11%), with or without EGF	In vitro: tympanic membrane cell migration and viability In vivo: implantation of CS in tympanic perforations; endoscopic and HT analyses Control: untreated perforations Sample: *n* = 25	In vitro: CS+EGF demonstrated greater cell migration and viability than pure CS In vivo: CS+EGF promoted better closure of perforations and better regeneration than the control	CS+EGF produced favorable results in vitro and in vivo with the regeneration of tympanic perforations (*P* < 0.05), being a potential alternative to surgery

Zhou et al. 2014 [34]	Vascular tissue	CS+PCL (ratio NA) in the form of tubules combined with endothelial cells MW and DA: NA	In vitro: CC in culture of endothelial cells In vivo: implantation in vascular defects in the carotids of dogs; western-blot and RT-PCR analyses Sample: *n* = 6	CS+PCL showed cytocompatibility in vitro; CS+PCL+ endothelial cells promoted normal vascular flow and endothelial regeneration in vivo	CS+PCL impregnated with endothelial cells were effective in vascular tissue regeneration

Zang et al. 2014 [33]	Periodontal ligament	CS (medium MW and DA 15–25%) in the forms of powder or solution	In vitro: CC (periodontal ligament cells) and physicochemical analyses In vivo: insertion of gel in furcation lesions in dogs' teeth; HT analysis Negative control group without CS Sample: *n* = 8	The hydrogel obtained with autoclaved CS in the form of powder exhibited the filling of 80% of the defects. Bone and periodontal regeneration was effective after 12 weeks (*P* < 0.05)	The autoclaving of CS in the form of powder did not change its physicochemical properties; CS was effective in the regeneration of furcation lesions

Jiang et al. 2015 [90]	Periodontal ligament	CS, whether or not combined with PCE MW and DA not reported	In vitro: CC (rat BMMSCs) and expression of periodontal ligament markers; In vivo: insertion in periodontal defects with BiOss® in rats; HT, immunofluorescence, and micro-CT analyses; negative control without scaffold; sample: *n* = 5	CS and CS+PCE promoted periodontal regeneration, with greater organization of fibers in the CS+PCE group after 8 weeks (*P* < 0.05)	CS scaffolds with PCE nanofibrils were found to have great potential for application for regeneration of periodontal ligament

Denost et al. 2015 [26]	Colorectal tissue	CS membranes with two types of hydrogel (DA 98.5% and 97%; MW 420 and 487 kDa, resp.)	In vitro: CC and cell differentiation (human adipose-derived stem cells) In vivo: implantation in lesions in the intestines of rabbits; macroscopic and HT analyses Sample: *n* = 4	In vitro: CS and control (commercial membrane) were cytocompatible In vivo: CS exhibited lower (*P* > 0.05) degree of inflammation and complete colorectal regeneration after 8 weeks	Membranes in multiple layers of CS demonstrated potential in colorectal regeneration, suggesting better results than with the commercial material

CC: cytocompatibility; CS: chitosan; CT/micro-CT: computed tomography/micro-computed tomography; DA: degree of acetylation; EGF: epithelial growth factor; HT: histological; kDa: kilodaltons; MW: molecular weight; PCE: polycaprolactone-polyethylene glycol polymer; PCL: polycaprolactone; PCR: polymerase chain reaction; SF: silk fibroin.
